# Continuous blood glucose monitoring reduces the risk to ICU patients

**DOI:** 10.1186/cc14449

**Published:** 2015-03-16

**Authors:** KP Mulavisala, J Norrie, B Crane, N Barwell

**Affiliations:** 1 CARE Hospitals, Hyderabad, India; 2 SumStats Ltd, Edinburgh, UK; 3 GlySure Ltd, Abingdon, UK

## Introduction

GlySure Limited (Abingdon, UK), has developed a continuous intravascular glucose monitoring system (CIGMS) to simplify the application of hospital protocols for optimal glucose control at the point of care. We have previously reported on the early

## Results

achieved in cardiac patients [1] and MICU patients [2]. This initial success has been sustained and demonstrated in further patient groups. We have now reached the point where we can conjecture upon the regular application of the GlySure CIGMS in day-to-day ICU practice. This in turn prompts the question, 'How effective will continuous blood glucose data prove in such routine use?' Using actual case data, we have shown how comparing the mean absolute relative difference (MARD) and integration of the area under the curve (AUC) from the continuous glucose monitoring and intermittent measurement can be used to measure patient risk.

## Methods

The analysis used aggregated case data generated from our recent clinical trials, where a GlySure sterile, single-use sensor and dedicated monitoring system was used to measure the blood glucose concentration in patients continuously and in real time. The measurement of risk was compared using the MARD, an accepted error calculation tool, and the AUC was calculated using an AUC analysis software program.

## Results

When MARD from the GlySure sensor and intermittent measurement using the hospital's existing protocol was compared, the measure of risk to the patient (that is, the uncertainty regarding the patient's absolute blood glucose status) for the GlySure sensor was 50.5% lower than the intermittent measurement. The results also showed that as the variability of the BG data increases, the benefit of continuous monitoring increases by significantly reducing patient risk. The continuous monitoring reduces the patient's risk by 88%, 73%, and 69% respectively in high, medium and low variability situations.

## Conclusion

It is more and more evident that continuous glucose technology will be instrumental in driving safe and effective glucose management protocols that will support more consistent glycemic management standards within ICUs and across institutions.
